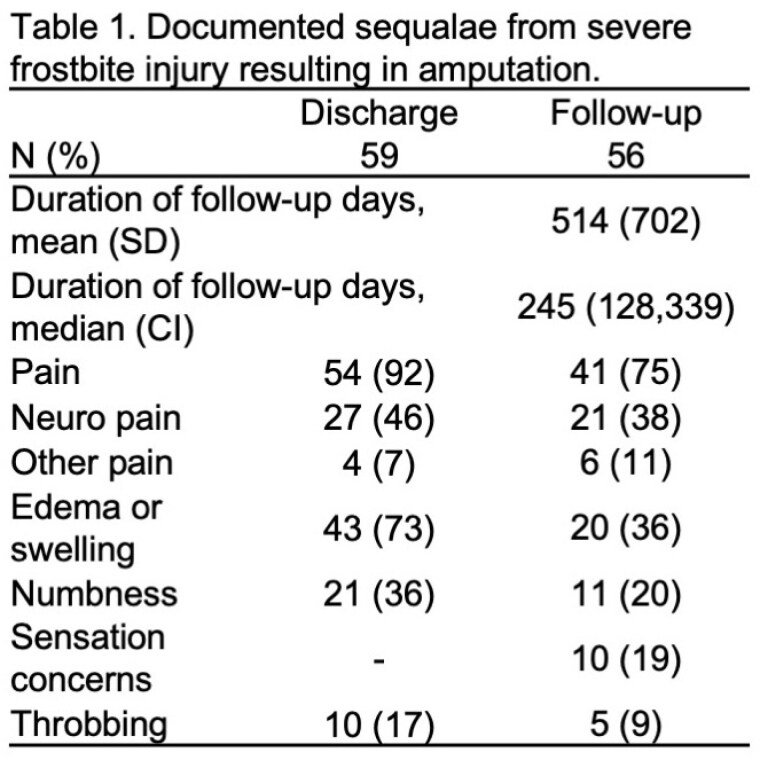# 696 Long-Term Functional Impairments and Rehabilitation Needs of Severe Frostbite Injury Resulting in Amputation

**DOI:** 10.1093/jbcr/iraf019.325

**Published:** 2025-04-01

**Authors:** Lexy Kindt, Charly Vang, Lisa Nelson, Sarah Zemek, Jana Almendinger, Patricia White, Rediat Tilahun, Emily Colonna, Kyle Schmitz, Derek Lumbard, Rachel Nygaard

**Affiliations:** Hennepin County Medical Center; Hennepin Health Care Research Institute; Hennepin Healthcare; Hennepin Healthcare; Hennepin County Medical Center; Hennepin County Medical Center; Hennepin Health Care Research Institute; Hennepin Healthcare; Hennepin Healthcare; Hennepin Healthcare; Hennepin Healthcare

## Abstract

**Introduction:**

Severe frostbite injury can lead to long-term physical disability, particularly in patients who require amputation. While interventions often focus on limb salvage, understanding long-term functional outcomes is crucial for planning rehabilitation and improving post-amputation care. This study aimed to evaluate the functional status, pain levels, and disability in patients with severe frostbite.

**Methods:**

This retrospective study included 59 severe frostbite injured patients with amputations. Charts were reviewed at discharge, subsequent hospitalizations, and at follow-ups. Outcome measures included disability (issues with balance, grip, or range of motion), requiring assistance or modifications for activities of daily living (ADLs), ambulation status (independent, assist-device, non-ambulatory), and sequelae from the injury (presence of pain, edema, numbness, throbbing and pain medication). Outcomes at follow-up included data from the longest follow-up visit with non-missing data.

**Results:**

At discharge, 70% of lower extremity injury and 76% of upper extremity injury reported disability with 58% requiring ADL assistance. Ambulation assistance was required for 37% of patients and 15% were non-ambulatory. None of the patients were able to return to work, however 68% were unemployed prior to the injury. Most patients had one procedure (48%), 22% had 2, 14% had 3, and 16% had 4-7 procedures. Sequelae from the injury is documented in Table 1. The follow-up time ranged from zero (lost to follow-up) to 9.6 years [mean 514 days (SD 702) and median 245 days days (95% CI 128, 339)]. At follow-up, disability was reported in 33% of lower extremity injury and 51% of upper extremity injury. Most required no assistance with ADLs (80%), however 23% required ambulation assistance devices. At follow-up return to work occurred in 16% of patients. Long-term sequelae is detailed in Table 1.

**Conclusions:**

Few studies evaluate long-term functional outcomes and disability in patients with severe frostbite who require amputation, providing crucial insights for improving post-amputation care. Severe frostbite injuries result in substantial long-term functional impairments, particularly in ambulation and ADLs. Most patients experience persistent pain, numbness, and other complications. These findings underscore the need for comprehensive rehabilitation programs to enhance recovery and quality of life for severe frostbite survivors.

**Applicability of Research to Practice:**

Rehabilitation efforts focusing on improving ambulation, upper extremity function, and managing chronic neurovascular symptoms should be prioritized for patients recovering from severe frostbite injuries.

**Funding for the Study:**

N/A